# Effect of Subhypnotic Dose of Propofol on Respiratory Adverse Events Following Postoperative Tonsillectomy/Adenotosillecomy: A Systematic Review and Meta-Analysis

**DOI:** 10.3390/jcm15052074

**Published:** 2026-03-09

**Authors:** Noof Albannai, Abdullah Esmaeel, Dana Alsaif, Fajer Alabdulrazzaq, Salman Alshammari, Abdullah M. Alharran, Ebrahim Almulla, Shawkat Abdulrahman

**Affiliations:** 1 School of Medicine, Trinity College Dublin, D02 PN40 Dublin, Ireland; esmaeela@tcd.ie (A.E.);; 2 Kuwait Institute for Medical Specialization, Kuwait City P.O. 1793, Kuwaitff.alabdulrazzaq@moh.gov.kw (F.A.); 3 College of Medicine and Medical Sciences, Arabian Gulf University, Manama 329, Bahrain; 4 Department of Otolaryngology, Head and Neck Surgery, Tallaght University Hospital, D24 NR0A Dublin, Ireland

**Keywords:** pediatric, children, tonsillectomy, adenoidectomy, extubation, propofol, sub-hypnotic dose, meta-analysis, laryngospasm

## Abstract

**Background**: Laryngospasm is defined as glottis closure due to reflex constriction of the laryngeal muscles. It is one of the most common complications following pediatric anesthesia that can lead to hypoxemia, bradycardia, or aspiration. Laryngospasm following tracheal extubation has different reasons: presence of secretions, foreign body in the airway, or pain at the site of surgery. Propofol is usually used as an induction or maintenance agent. However, its use with the subhypnotic dose (0.5 mg/kg) is increasing nowadays for reducing the incidence of laryngospasm. This systematic review and meta-analysis aim to assess the efficacy of subhypnotic propofol in reducing the incidence of laryngospasm and respiratory complications in children following tonsillectomy or adenotonsillectomy and before extubation. **Methods**: We systematically searched the following databases: PubMed, Cochrane Library, Scopus, and Web of Science. Studies were included if they used propofol with a low dose (0.5 mg/kg) following tonsillectomy and before extubation. Both Randomized Controlled Trials (RCTs) and cohort studies published up until 27 December 2025 were included. We used the R software for statistical analysis. We employed a random-effects model for the analysis. Continuous outcomes were analyzed as mean differences (MD) and dichotomous data as risk ratios (RR), with 95% confidence intervals (CI). Heterogeneity was assessed using I^2^ statistics. **Results**: Our review included four RCTs and one prospective cohort study with 593 participants. Our analysis showed that propofol was significantly associated with a low incidence of laryngospasm (RR = 0.25, 95% CI 0.13–0.49), cough (RR = 0.08, 95% CI 0.01–0.62), and agitation (RR = 0.15, 95% CI 0.03–0.72) compared with the control group. However, there were no significant differences regarding laryngeal occlusion (RR = 0.70, 95% CI 0.20–2.46), cyanosis (RR = 1.13, 95% CI 0.14–9.43), stridor (RR = 1.38, 95% CI 0.76–2.50), and the duration of surgery (MD = 1.81, 95% CI −0.74 to 4.36). **Conclusions**: Our findings state that propofol had a lower significant incidence of laryngospasm than the control. Trial sequential analysis for laryngospasm indicated that evidence is conclusive. However, regarding the other outcomes, the evidence is still inconclusive, which suggests the need for future large-scale RCTs with larger sample sizes to validate these findings.

## 1. Introduction

The emergence from anesthesia and tracheal extubation represent a critical period in pediatric anesthesia, during which a significant proportion of serious airway complications occur [1,2]. These events, ranging from coughing and agitation to life-threatening laryngospasm, are often triggered by stimuli such as secretions or blood in the airway, posing unique challenges in children due to their smaller, more reactive airways [3,4]. Having small and reactive airways, a high or more anterior larynx, and increased sensitivity of laryngeal and bronchial reflexes make children more susceptible to respiratory complications [5].

Laryngospasm is one of the perioperative respiratory adverse events (PRAEs), which are most common following pediatric surgeries [6]. It is defined as an exaggerated and involuntary contraction of laryngeal muscles resulting in sustained closure of the vocal cords [7]. Laryngospasm is clinically graded in a four-point scale according to the severity, ranging from 0, no laryngospasm; 1, mild, which presents with inspiratory stridor; 2, moderate, which manifests as temporary total occlusion of the vocal cord; and 3, severe, where sustained obstruction and severe hypoxemia and cyanosis are present [8]. Several factors contribute to increasing the risk of developing laryngospasm, including congenital anomalies and upper respiratory tract infections (URTI), inappropriate deep anesthesia, intubation, and tonsillectomy and adenoidectomy [7]. Pharyngeal and laryngeal surgeries have an incidence of laryngospasm of 21–26% [9].

Laryngospasm is a potentially life-threatening condition that can lead to hypoxia, bradycardia, or cardiac arrest [3]. Therefore, variable modes of treatment are established, starting with the removal of the stimulus with administration of 100% oxygen and applying airway maneuvers, e.g., “Jaw Thrust” and “Larson’s maneuver” [9]. If there is no response, the patient is ventilated with a mask, and continuous positive airway pressure (CPAP) should be applied. The patient is shifted to intravenous injection of muscle relaxant succinylcholine 0.1 mg/kg concurrently with atropine to prevent bradycardia. Tracheal intubation will be the last option if the patient still has laryngospasm [3].

Propofol, an intravenous (IV) anesthetic that acts on GABA-A receptors, is known by its role in depressing airway reflexes [10]. It is usually used for induction and maintenance therapy with a dose of 2.5–3 mg/kg IV. The duration of action after a single bolus is approximately five to 10 min [8,11]. However, its effect on airway reflexes has generated a hypothesis of its use not just as an induction agent, but as a prophylactic measure [12]. Specifically, the administration of a subhypnotic dose (e.g., 0.5 mg/kg) prior to extubation is hypothesized to provide sufficient suppression of laryngeal reactivity to prevent laryngospasm while avoiding the prolonged sedation and respiratory depression associated with higher, hypnotic doses [8,11,13,14,15].

Though it is not involved in the guidelines, some clinical trials show a possible effect in decreasing the incidence of laryngospasm [8,11,13,14,15]. Therefore, we conducted our systematic review and meta-analysis to evaluate its efficacy in reducing the incidence of laryngospasm and other respiratory complications in children undergoing tonsillectomy or adenotonsillectomy.

## 2. Methods

### 2.1. Study Registration

The study protocol was registered on PROSPERO (CRD420261282682) on 14 January 2026. The registration was completed retrospectively after the completion of the literature search. This systematic review adhered to the Preferred Reporting Items for Systematic Reviews and Meta-Analyses (PRISMA) statement standards [16] and was conducted in accordance with the guidelines of the Cochrane handbook for systematic reviews [17]. PRISMA checklist is provided in the Supplementary Materials.

### 2.2. Literature Search and Study Selection

We searched the following databases: PubMed, Cochrane Library, Scopus, and Web of Science up to 27 December 2025 using search terms related to “children”, “tonsillectomy”, and “propofol”. Detailed search strategies are displayed in Table S1.

Then, we used EndNote 20 software (Clarivate Analytics, PA, USA) to remove duplicates [18]. We uploaded the retrieved references on the Rayyan website [19], and two independent authors conducted a two-step screening: the first step consisted of screening the titles/abstracts, and the second step consisted of screening the full-text articles of the identified abstracts for final eligibility. Any conflict was resolved by the third author’s decision.

### 2.3. Eligibility Criteria

We included studies that met the following PICO criteria:

- **Population:** Children who underwent tonsillectomy or adenotonsillectomy.
- **Intervention:** Propofol alone with subhypnotic dose (0.5 mg/kg) at the end of tonsillectomy or adenotonsillectomy before extubation. Studies that used propofol only as an induction or maintenance anesthetic drug with doses higher than 0.5 mg/kg were excluded.
- **Control:** Placebo (normal saline) or standard anesthetic care/lidocaine without the specific propofol bolus.
- **Outcomes:** Primary outcome was incidence of laryngospasm, while the secondary outcomes were any of the following: other respiratory complications, agitation, nausea, and vomiting.
- **Study design:** Randomized controlled trials (RCTs) and cohort studies were included.

We excluded the previous reviews, cross-sectional studies, single-arm studies, case reports, case series, conference abstracts, fine element studies, in vitro studies, animal studies, editorials, non-English studies, and non-peer-reviewed studies.

### 2.4. Data Extraction

Two independent authors conducted the data extraction using a standardized Excel sheet. The standardized sheet included three domains:

- Study characteristics including: study design, condition, drug of the intervention and the dose, number of patients, induction and maintenance anesthetic drugs, inclusion criteria, and primary outcomes.
- Baseline characteristics of the included study population, including: number of participants, age, male participants, weight, duration of surgery, and American Society of Anesthesiologists (ASA) physical status.
- Measurements of the studied outcomes.

### 2.5. Quality Assessment

Two independent authors evaluated the risk of bias in included studies using the Cochrane Risk of Bias assessment tool (ROB2), which encompassed five domains: randomization process, concealment of the allocation sequence, deviations from the intended interventions, missing outcome data, measurement of the outcome, selection of the reported results, and overall risk of bias [20]. For the cohort study, we used the Newcastle–Ottawa Scale to assess selection, comparability, and outcome [21]. Any conflicts were resolved by a third author. The RobVis web tool was used to create quality assessment figures [22].

### 2.6. Statistical Analysis

We used R 4.5.0 [23] with R Studio 2024.12.1+563 [24]. Continuous outcomes were pooled as mean differences (MDs) with corresponding 95% confidence intervals (CIs), while dichotomous outcomes were analyzed using risk ratios (RRs) with 95% CIs. Visual inspection of the forest plot was used to measure statistical heterogeneity between trials, in addition to I-squared (I2) and chi-squared (Chi2) statistics. I2 values of 50% indicated significant heterogeneity. All results were analyzed using a random-effects model. We utilized the Egger test [25] and funnel plot to assess publication bias and small-study effects where there were ten or more studies; otherwise, we used the Doi plot and LFK index [26]. We also used the trim-and-fill method to estimate the number of missing studies and update the pooled estimates [27].

Trial Sequential Analysis (TSA) was conducted to control the risks of random errors due to repeated significance testing in cumulative meta-analysis. We used Trial Sequential Analysis Viewer (TSA Viewer), a computer program (Version 0.9.5.10 Beta. Copenhagen: Copenhagen Trial Unit, Centre for Clinical Intervention Research, Rigshospitalet. 2016) [28]. The analysis was performed retrospectively, and study order was determined according to their appearance in the dataset.

## 3. Results

### 3.1. Literature Search

**Figure 1:**
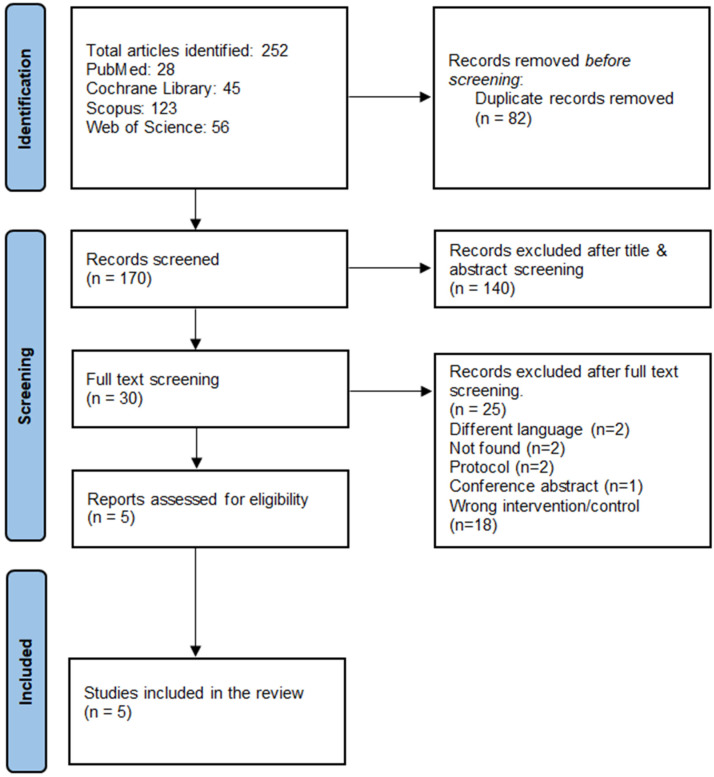
PRISMA flow chart for the systematic search and selection process.

Our systematic search through the different databases retrieved 252 articles, and after removing the duplicates, there were 170 eligible for the title and abstract screening. Then, thirty articles were eligible for the full-text screening, and finally, four RCTs [8,11,13,15] and one prospective cohort were included [14]. PRISMA flow diagram is shown in Figure 1.

### 3.2. Study Characteristics

Four RCTs and one prospective cohort study were included in our meta-analysis with a total of 593 children who underwent elective tonsillectomy. Most of them were males (58.7%). Their age ranged from two to fourteen years old. Children with a history of URTI, asthma, and steroid use were excluded in most studies. Most of them were of class I or II of the ASA physical status. Three studies allocated patients to either propofol 0.5 mg/kg or normal saline [8,14,15], one study allocated them to propofol or high-flow oxygen at a rate of >10 liters per minute [11] and the last one allocated them to propofol and lidocaine [13]. Regarding the induction anesthetic drug, thiopentone was used in two studies [14,15], propofol 2.5 mg/kg for two studies [8,11], and a combination of Midazolam 0.05 mg/kg, fentanyl 1 µg/kg, propofol 2 mg/kg, and atracurium 0.5 mg/kg for the last one [13]. (Table 1 and Table 2).

### 3.3. Quality Assessment

**Figure 2:**
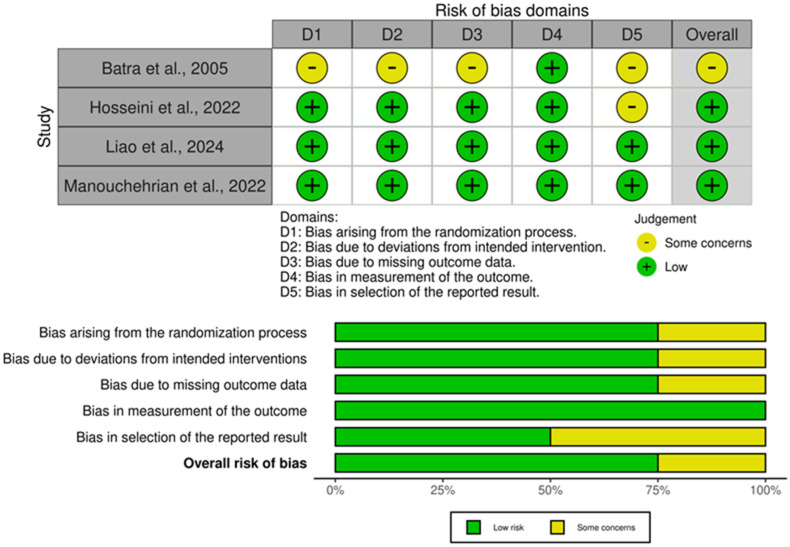
Summary and traffic light plots of the risk of bias of the included randomized controlled trials [8,11,13,15].

Regarding the four RCTs, all studies showed low risk regarding the measurement of the outcome. Also, three studies showed low overall risk of bias [8,11,13], while the last was of some concerns [15] (Figure 2). For the prospective cohort, it had a total score of seven, “good quality” regarding the domains [14] (Table S2).

### 3.4. Safety and Efficacy Outcomes

#### 3.4.1. Primary Efficacy Outcomes

**Laryngospasm**

**Figure 3:**
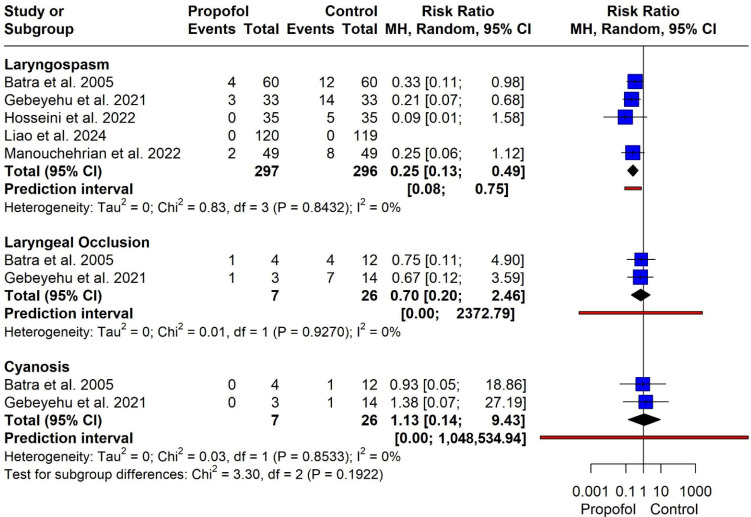
Forest plot for laryngospasm, laryngeal occlusion and cyanosis [8,11,13,14,15].

Five studies (*n* = 593 participants) were included. Use of propofol was associated with a significantly lower risk of laryngospasm compared with control (RR = 0.25, 95% CI 0.13–0.49). No statistical heterogeneity was observed (I^2^ = 0%, χ^2^ = 0.83, *p* = 0.84). The prediction interval did not cross the line of no effect (0.08–0.75) (Figure 3).

**Figure 4:**
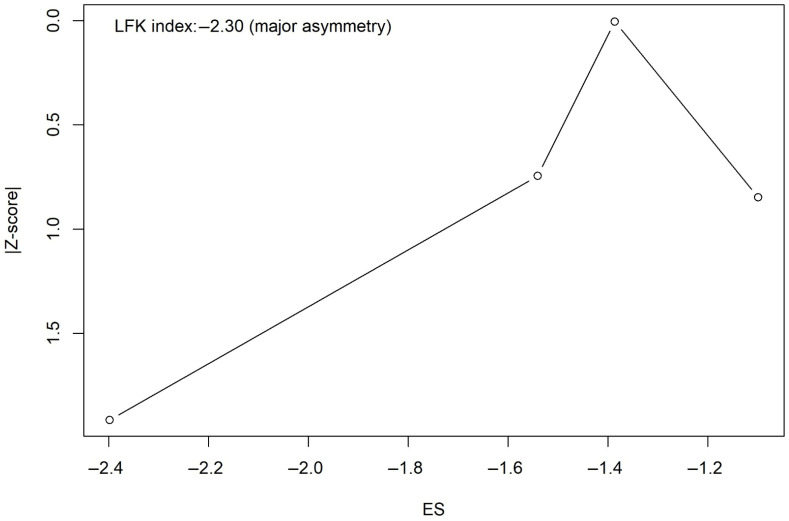
Doi plot for laryngospasm. Asymmetry in the plot as indicated by an LFK index of −2.30 suggests the presence of publication bias or small-study effects [8,11,13,14,15].

**Figure 5:**
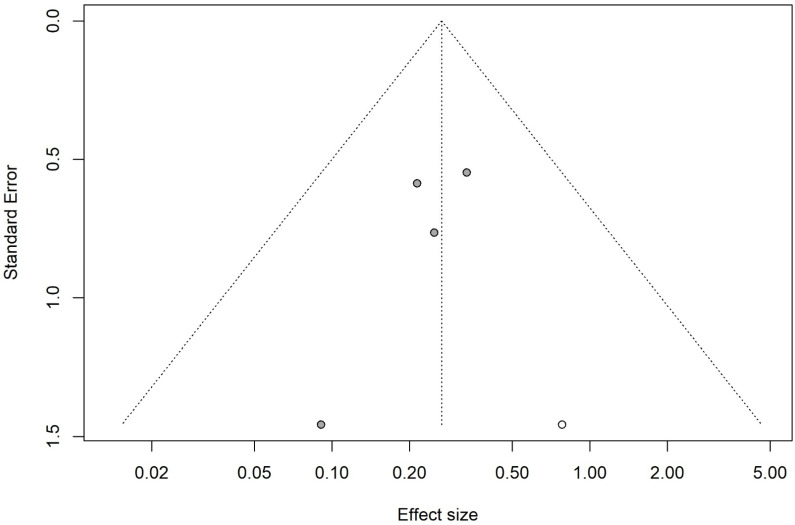
Funnel plot after trim and fill meta-analysis for laryngospasm. The imputed missing study (white circle) adjusts for the asymmetry observed in the Doi plot, providing a corrected pooled estimate [8,11,13,14,15].

Visual inspection of the Doi plot demonstrated marked asymmetry for the laryngospasm outcome, which was supported by a LFK index of −2.30, indicating major asymmetry (Figure 4). Therefore, the trim-and-fill method was applied, imputing one potentially missing study. After adjustment, the pooled analysis including five studies (424 participants; 212 in each group) showed that propofol was associated with a significantly reduced risk of laryngospasm compared with control (RR = 0.27, 95% CI 0.14–0.51; *p* < 0.0001). No statistical heterogeneity was observed following adjustment (I^2^ = 0%, Q = 1.40, df = 4, *p* = 0.84) (Figure 5).

**Figure 6:**
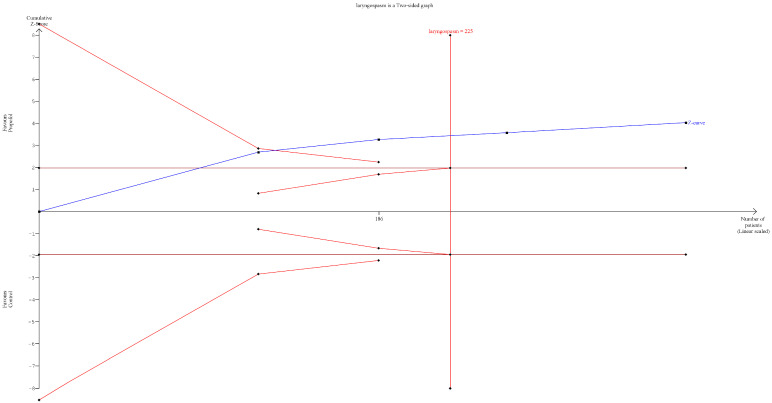
Trial sequential graph for laryngospasm. The cumulative Z-curve crosses both the conventional boundary and the trial sequential monitoring boundary, indicating that the required information size has been met [8,11,13,14,15].

Trial sequential analysis demonstrated that the cumulative Z-curve crossed the conventional boundary for statistical significance, indicating a significant reduction in laryngospasm with propofol compared with control. Because the Z-curve has crossed the monitoring boundaries, the graph suggests that the evidence may be conclusive. However, caution is needed while interpreting this result given the limited number of included studies. (Figure 6).

#### 3.4.2. Secondary Outcomes

**Laryngeal occlusion**

Two studies (*n* = 33 participants) were analyzed. There was no statistically significant difference between propofol and control (RR = 0.70, 95% CI 0.20–2.46). Heterogeneity was absent (I^2^ = 0%). The prediction interval was wide (0.00–2372.79), reflecting substantial uncertainty due to the small number of studies and events (Figure 3).

**Cyanosis**

Two studies (*n* = 33 participants) contributed data. No significant difference was observed between groups (RR = 1.13, 95% CI 0.14–9.43), with no detected heterogeneity (I^2^ = 0%). The prediction interval was extremely wide (0.00–1,048,534.94), indicating imprecision (Figure 3).

**Stridor**

**Figure 7:**
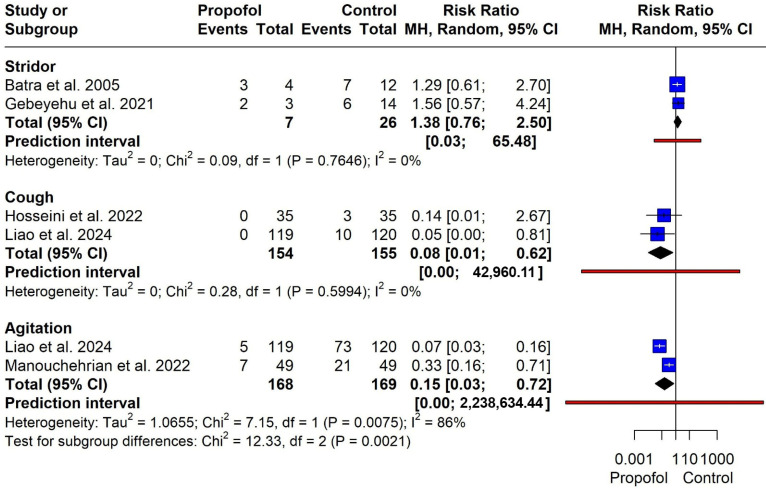
Forest plot for stridor, cough and agitation [8,11,13,14,15].

Two studies (*n* = 33 participants) were included. There was no statistically significant difference between the propofol and control groups (RR = 1.38, 95% CI 0.76–2.50). No heterogeneity was detected (I^2^ = 0%, χ^2^ = 0.09, *p* = 0.76). The prediction interval was wide (0.03–65.48), indicating substantial uncertainty due to the limited number of studies and events (Figure 7).

**Cough**

Two studies (*n* = 309 participants) were analyzed. Propofol was associated with a significantly reduced risk of cough compared with control (RR = 0.08, 95% CI 0.01–0.62). No heterogeneity was observed (I^2^ = 0%, χ^2^ = 0.28, *p* = 0.60). The prediction interval was wide (0.00–42,960.11), reflecting imprecision despite the statistically significant pooled effect (Figure 7).

**Agitation**

Two studies (*n* = 337 participants) contributed data. Propofol significantly reduced the risk of agitation compared with control (RR = 0.15, 95% CI 0.03–0.72). However, substantial heterogeneity was observed (I^2^ = 86%, χ^2^ = 7.15, *p* = 0.0075). The prediction interval was wide (0.00–2,238,634.44), indicating variability in effect estimates across studies (Figure 7).

**Duration of surgery (minutes)**

**Figure 8:**
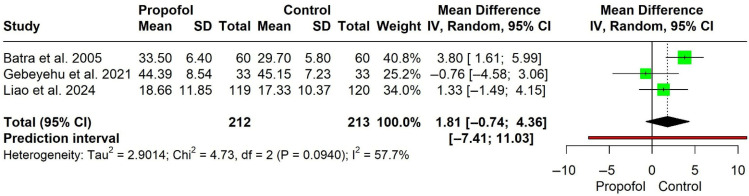
Forest plot for duration of surgery (minutes) [11,14,15].

Three studies involving 425 participants (212 in the propofol group and 213 in the control group) were included in the analysis. There was no statistically significant difference between propofol and control groups (MD = 1.81, 95% CI −0.74 to 4.36). Moderate heterogeneity was observed (I^2^ = 57.7%, χ^2^ = 4.73, df = 2, *p* = 0.094). The prediction interval was wide (−7.41 to 11.03), indicating considerable uncertainty in the expected effect of future studies (Figure 8).

## 4. Discussion

### 4.1. Summary of Findings

Propofol was significantly associated with a low incidence of laryngospasm compared with the control group (RR = 0.25). Although the results showed major asymmetry when using the Doi plot and LFK index of −2.3, which may be attributed to some publication bias or a small number of participants, propofol still has a lower significant incidence for laryngospasm development than the control (RR = 0.27, *p* < 0.0001). Propofol also has a narrow prediction interval, which strengthens our results. Based on the available data, the evidence for laryngospasm is promising, though the small number of studies and variability in laryngospasm grading across trials warrant some caution in interpreting these findings. Regarding our secondary outcomes, propofol significantly reduced the risk of cough and agitation compared with the control group. There were no statistically significant differences between propofol and the control regarding laryngeal occlusion, cyanosis, stridor, and the duration of surgery. All our secondary outcomes had a wide prediction interval with a significant heterogeneity found in agitation and the duration of stay, which suggests the need for future RCTs with a large-scale sample size.

### 4.2. Interpretation of Findings

Otolaryngology surgeries and tracheal intubation are associated with a higher risk for the development of PRAE in pediatric patients [29]. All studies included in this systematic review included children with ASA physical status I to III, which limits the influence of comorbid conditions as potential confounders [30]. Patients with ASA physical health higher than III were at a higher risk of getting PRAE than others (adjusted odds ratio (AOR): 5.2, CI: 1.9–22.9) [31]. This highlights the need for perioperative strategies aimed at modulating airway reflexes during emergence, particularly in high-risk procedures such as tonsillectomy.

For prevention of laryngospasm and controlling such reflexes, current evidence sets the priority for non-pharmacological strategies, such as depth of anesthesia, airway clearance, airway maneuvers, and muscle relaxants, when needed [32]. They are effective for low to moderate cases with partial laryngospasm. In contrast, pharmacological treatments are definitive treatment for moderate to severe cases [33]. Clinical trials used different medications such as propofol, lidocaine 1–2 mg/kg, and magnesium sulphate to assess their efficacy [8,34]. However, until now, there is no recommendation or standard guideline for pharmacological agents to prevent the reflex-mediated complications [35].

The doses of propofol vary in the literature according to its use. The typical induction dose for pediatric general anesthesia is 2.5–3 mg/kg, which causes loss of consciousness within 30–60 s [36]. In contrast, most clinical trials using subhypnotic propofol for preventive measures have used 0.5 mg/kg [8]. Regarding lidocaine, it is typically given either intravenously or topically. Lidocaine acts both centrally by causing depression of brainstem reflexes and peripherally through blocking sensory input from the larynx and pharynx to the CNS, causing central reflex suppression as a result [37]. Compared with placebo, lidocaine could significantly decrease the incidence of laryngospasm (OR 0.50; 95% CI 0.27 to 0.95; *p* = 0.033) and other PRAE [38]. However, the evidence for propofol versus lidocaine is limited [13]. Also, according to a systematic review, the small dose of dexmedetomidine 0.4 to 0.5 μg/kg seemed to be safer and more effective, causing smooth extubation and no laryngospasm, than the large dose of higher than 0.5 μg/kg, which caused bradycardia, hypotension, and sedation [33].

Timing to give propofol is a major concern and is a main reason for heterogeneous results. Subhypnotic propofol 0.5 mg/kg given before extubation has mainly a preventive role where airway reflexes are strongest during emergence, and propofol use will suppress laryngeal and cough reflexes and emergence agitation. Consistent with all articles included in this systematic review, propofol was given before extubation and showed significant and promising evidence for reducing the incidence of laryngospasm [8,14,15]. In contrast, administration after extubation is mainly used as a rescue strategy to attenuate established agitation or coughing and appears less reliable for preventing laryngospasm [39,40]. The effect of timing is also relevant to lidocaine [41].

In this meta-analysis, propofol could significantly reduce the risk for laryngospasm (RR = 0.25), cough (RR = 0.08), and agitation (RR = 0.51) following tonsillectomy. This consistently matches the data previously published [8]. Another meta-analysis proved that using propofol with a bolus dose in the induction of anesthesia had a significantly lower odds ratio (OR) than sevoflurane induction for having PRAE (OR = 0.35), laryngospasm (OR = 0.17), and airway obstruction (OR = 0.32) [35]. In obstetric patients, a low dose of propofol of 0.5 mg/kg could decrease the incidence of laryngospasm more than lidocaine [4]. The impact of a pre-extubation propofol bolus on time to awakening is a crucial clinical consideration. In contrast to a full induction dose, it is hypothesized that the subhypnotic dose of 0.5 mg/kg is unlikely to result in meaningful emergence delays. In two of the included studies, awakening times were reported and no statistically significant delay was identified when propofol was administered [11,13]. Nonetheless, more investigation using standardized awakening metrics is necessary.

Our results suggest no difference between propofol and placebo regarding different grades of laryngospasm, including stridor, laryngeal occlusion, and cyanosis. This finding should be interpreted with caution, as grading of laryngospasm severity was inconsistently reported across the included studies, limiting the ability to perform a robust comparative analysis.

The significant heterogeneity found regarding the agitation could be attributed to several methodological and clinical factors, including the variation in maintenance anesthetic drug, the different control group in both studies (lidocaine [13] versus normal saline [11]), and relatively different sample sizes. Similarly, the heterogeneity related to the duration of surgery may be explained by the different induction anesthetic drugs among studies and the inclusion of a prospective cohort study [14].

### 4.3. Strengths and Limitations

This study is the first systematic review and meta-analysis to assess the relationship between the use of subhypnotic propofol (0.5 mg/kg) and laryngospasm, highlighting its protective effect on laryngospasm incidence when propofol is administered before extubation. This study had rigorous methodology with adherence to PRISMA guidelines. The use of the random-effects model and assessment of small-study effects via Doi and trim and fill methods provided a transparent assessment of effect size and consistency. However, our study has several limitations. The inclusion of a prospective cohort acts as a source of bias compared with RCTs [14]. The absence of design-specific sensitivity analyses, such as analyses restricted to RCTs, should be acknowledged. The number of eligible clinical trials was limited, and substantial clinical and methodological heterogeneity was observed for some of the secondary outcomes: agitation (I^2^ = 86%) and the duration of surgery (I^2^ = 57.7%). Furthermore, the evidence of marked asymmetry in Doi/funnel plots and high LFK indices, together with the need for trim-and-fill adjustments, materially altered effect sizes for the primary outcome “laryngospsam”. The wide prediction intervals for secondary outcomes indicate uncertainty regarding the magnitude of effect in future settings, and for outcomes like agitation and cough, the substantial statistical heterogeneity further limits confidence in the pooled estimate.

Furthermore, the included studies did not provide individual patient-level data, preventing a pooled analysis of the demographic or anesthetic characteristics of children who developed laryngospasm Additionally, PROSPERO registration was completed in a retrospective manner, which might be a potential limitation to our findings.

### 4.4. Clinical Implications and Future Recommendations

Our findings determined the protective effect of subhypnotic propofol dose 0.5 mg/kg in reducing the incidence of laryngospasm in children undergoing tonsillctomy. It also highlights the importance of timing, particularly administration before extubation. The results for laryngospasm after trial sequential analysis provide evidence that the available data are supportive regarding the preventive use of propofol for this outcome. However, given the limitations discussed, future trials could still provide valuable information on optimal dosing, timing, and patient subgroups. Also, studies with large scales and large sample sizes are needed to determine the efficacy of low-dose propofol on agitation, duration of surgery, cough, and stridor. Additionally, future studies addressing propofol with different dose regimens will help to define the optimal dose for pediatric anesthetic complications. Furthermore, future research should aim to directly compare the success rate of propofol alone versus in combination with other strategies (e.g., positive pressure ventilation, Larson maneuver) for the acute management of established laryngospasm.

## 5. Conclusions

This systematic review and meta-analysis states that propofol with a low dose of 0.5 mg/kg had a significantly lower incidence of laryngospasm than the control. The current evidence is promising based on trial sequential analysis, though the limited number of studies means some uncertainty remains. Timing for administration of propofol is critical: propofol given before extubation acts as a preventive measure, whereas administration after extubation serves a curative role. Regarding agitation, duration of surgery, cough, and stridor, the evidence is still inconclusive due to wide prediction intervals and significant heterogeneity. Future large-scale RCTs with larger sample sizes are needed to optimize dosing and timing strategies for safe and effective airway management in pediatric anesthesia.

## Figures and Tables

**Table 1:** Summary of study characteristics.

**Study ID**	**Study Design**	**Country**	**Total Sample Size**	**Population**	**Inclusion Criteria**	**Induction Anesthesia**	**Maintenance Anesthesia**	**Intervention**	**Control**	**Primary** **Outcomes**
Batra et al. 2005 [15]	RCT, double-blind	Kuwait	120	ASA I–II, 3–14 years, elective tonsillectomy/adenoidectomy	ASA I–II, 3–14 years, elective T&A. Excluded: recent URI, asthma, difficult airway	Thiopental 4–5 mg/kg or sevoflurane; suxamethonium 1.5 mg/kg for intubation	Isoflurane 1.5–2.5% in 66% N_2_O/O_2_	Propofol 0.5 mg/kg 60 s before extubation	Normal saline	Laryngospasm (stridor, occlusion, cyanosis)
Gebeyehu et al. 2021 [14]	Prospective cohort	Ethiopia	66	Up to 9 years, elective adenotonsillectomy	Elective T&A; excluded: recent URTI, asthma, difficult airway, propofol induction	Thiopental or ketamine; suxamethonium 1–2 mg/kg	Halothane or isoflurane in O_2_	Propofol 0.5 mg/kg 60 s before extubation	Routine practice (no propofol)	Laryngospasm (stridor, occlusion, cyanosis)
Hosseini et al. 2022 [8]	RCT, double-blind	Iran	70	ASA I, 2–12 years, elective tonsillectomy	ASA I, 2–12 years; excluded: recent URI, steroids, airway disorders, >2 intubation attempts	Propofol 2.5 mg/kg + atracurium 0.5 mg/kg	Isoflurane 1% + 50% N_2_O/O_2_	Propofol 0.5 mg/kg before extubation	Normal saline	Laryngospasm, cough, nausea
Liao et al. 2024 [11]	RCT, double-blind	China	239	ASA I–III, 3–8 years, elective T&A	ASA I–III, 3–8 years; excluded: cardiac/pulmonary/neurological disease, tumors	Propofol 3 mg/kg + remifentanil 3–5 µg/kg + atropine	Sevoflurane 2.5% + 50% N_2_O/O_2_	Propofol 0.5 mg/kg repeated (total 1–2 mg/kg) before extubation	Saline 0.15 mL/kg + high-flow O_2_	PRAEs: laryngospasm, bronchospasm, breath-holding, severe cough, desaturation, airway obstruction
Manouchehrian et al. 2022 [13]	RCT, double-blind	Iran	98	ASA I–II, 3–14 years, elective tonsillectomy	ASA I–II, 3–14 years; excluded: cardiorespiratory disease, recent URI, allergies to propofol/lidocaine	Midazolam 0.05 mg/kg + fentanyl 1 µg/kg + propofol 2 mg/kg + atracurium 0.5 mg/kg	Isoflurane 1.2% + 50% N_2_O/O_2_	Propofol 0.5 mg/kg 2 min before extubation	Lidocaine 2% 1 mg/kg	Laryngospasm, inspiratory stridor, hemodynamics

Abbreviations: RCT: Randomized Controlled Trial, ASA: American Society of Anesthesiologists, URTI: Upper Respiratory Tract Infection, URI: Upper Respiratory Infection, T&A: Tonsillectomy and Adenoidectomy, PRAE: Perioperative Respiratory Adverse Event, N_2_O: Nitrous Oxide, O_2_: Oxygen.

**Table 2:** Baseline characteristics.

**Study ID**	**Study Group**	**Sample Size, *n***	**Age, Mean (SD)**	**Male, *n* (%)**	**Weight, Mean (SD)**	**ASA Physical** **Status (I/II/III)**	**Duration of** **Surgery (min), Mean (SD)**
Batra et al. 2005 [15]	Propofol 0.5 mg/kg	60	5.6 (2.5)	38 (63.3%)	24.5 (7.9)	46/14/0	33.5 (6.4)
	Normal saline	60	6.1 (2.7)	36 (60%)	26.7 (8.8)	48/12/0	29.7 (5.8)
Gebeyehu et al. 2021 [14]	Propofol 0.5 mg/kg	33	5.65 (2.572)	17 (51.5%)	21.1 (5.46)	32/1/0	44.39 (8.54)
	Routine practice	33	5.94 (2.499)	17 (51.5%)	21.18 (5.37)	31/2/0	45.15 (7.23)
Hosseini et al. 2022 [8]	Propofol 0.5 mg/kg	35	6.657 (2.566)	17 (48.57%)	24.826 (11.144)	All ASA I	Not reported
	Normal saline	35	7.171 (2.791)	19 (54.28%)	26.114 (1.687)	All ASA I	Not reported
Liao et al. 2024 [11]	Propofol 0.5 mg/kg	119	4.5 (1.33)	73 (61.3%)	19.77 (4.61)	77/40/2	18.66 (11.85)
	Normal saline	120	4.75 (1.49)	72 (60%)	20.39 (5.42)	81/36/3	17.33 (10.37)
Manouchehrian et al. 2022 [13]	Propofol 0.5 mg/kg	49	7.11 (2.9)	28 (57.1%)	23.8 (10.71)	All ASA I–II	Not reported
	Lidocaine 1 mg/kg	49	6.86 (2.96)	31 (63.3%)	25.14 (10.91)	All ASA I–II	Not reported

Abbreviations: ASA: American Society of Anesthesiologists, SD: Standard Deviation.

## Data Availability

Data available on request from the corresponding author.

## Supplementary Materials

The following supporting information can be downloaded at https://www.mdpi.com/article/10.3390/jcm15052074/s1, Table S1: Search strategies; Table S2: Newcastle–Ottawa Quality Assessment Scale (Cohort Studies); PRISMA checklist.
